# Dantrolene Administration in the Management of the Prehospital Patient with Methylenedioxymethamphetamine Overdose: A Case Series and Literature Review

**DOI:** 10.1155/2022/5346792

**Published:** 2022-08-27

**Authors:** Kia Nikoomanesh, Alexander T. Phan, Julian Choi, Sarkis Arabian, Michael M. Neeki

**Affiliations:** ^1^Department of Critical Care Medicine, Arrowhead Regional Medical Center, Colton, CA 92324, USA; ^2^California University of Science and Medicine, Colton, CA 92324, USA; ^3^Department of Internal Medicine, Arrowhead Regional Medical Center, Colton, CA 92324, USA; ^4^Department of Emergency Medicine, Arrowhead Regional Medical Center, Colton, CA 92324, USA

## Abstract

Methylenedioxymethamphetamine (MDMA) is a psychoactive substance that is used commonly as a recreational drug at rave music festivals. MDMA intoxication can cause a myriad of symptoms and side effects including the manifestation of hyperpyrexia in patients. Hyperpyrexia can mimic a heat stroke and ultimately lead to various forms of end-organ damage. The most common methods used in treating MDMA-induced hyperpyrexia focus on the rapid reduction of core body temperature. Various off-label medications have also been used in combating MDMA-induced hyperpyrexia. Dantrolene is one such medication, although its role in the treatment of MDMA intoxication remains uncertain. This case series preliminarily examines the efficacy of dantrolene in mitigating MDMA-induced hyperpyrexia and potentially reducing the risk of end-organ damage in patients suffering from MDMA overdose. This study focuses on nine patients who presented after ingesting various forms of MDMA at “rave” music events. All patients were found to be hyperthermic in the field with a maximum core body temperature of 109 degrees Fahrenheit. All patients were immediately managed by cooling measures, and seven patients additionally received dantrolene in the field before being transferred to Arrowhead Regional Medical Center. Upon arrival to the hospital, nearly every patient was found to have significantly decreased body temperatures when compared to previously measured body temperatures out in the field. However, nearly all patients in the study were also noted to have laboratory abnormalities consistent with various forms of end-organ damage. The degree and severity of end-organ damage observed in MDMA-induced hyperpyrexia seem to be a function of initial core body temperature. Higher core body temperature tends to correlate with more forms of end-organ damage and a higher severity of end-organ damage. Intervention with dantrolene and cooling measures appeared to have no effect on reducing the risk of developing end-organ damage in this patient population.

## 1. Introduction

Methylenedioxymethamphetamine (MDMA), also known as “ecstasy” or “molly,” is a psychoactive substance commonly abused at electronic dance music festivals, “raves,” dance clubs, and other social venues. MDMA is an amphetamine derivative that also has some pharmacological properties of mescaline. The amphetamine properties of MDMA amplify the user's sympathetic nervous system, while its mescaline properties provide both hallucinogenic and psychogenic effects. MDMA ingestion can lead to several side effects ranging from minor clinical symptoms to potentially fatal complications. These complications are grouped into four categories of toxicity: hepatic, cardiovascular, cerebral, and hyperpyrexic [1]. The hyperpyrexic pattern of toxicity has been hypothesized to be the most dangerous. It is believed to be the product of increased adrenergic, serotonergic, and dopaminergic responses in the body. The adrenergic response leads to peripheral vasoconstriction which in turn decreases heat dissipation. Meanwhile, increased serotonergic and dopaminergic effects disrupt the body's central hypothalamic thermoregulation and induce serotonin syndrome, which can thereby increase thermogenesis [2, 3]. These mechanisms induce hyperthermia, which is often exacerbated by overcrowded club and party settings where MDMA is taken [4, 5]. Patients thereby suffer from hyperpyrexia which can mimic a heat stroke, leading to rhabdomyolysis, disseminated intravascular coagulation (DIC), liver damage, and acute renal failure [2, 6].

Current standard methods of treating MDMA-induced hyperpyrexia include the use of supportive measures and active cooling techniques, such as chilled intravenous fluids, ice baths, and cooling blankets. In addition, multiple off-label medications have been trialed in the context of treating MDMA-induced hyperpyrexia. Dantrolene is one such medication. Over the years, many case reports and studies have investigated the efficacy of dantrolene in reducing mortality in patients with MDMA-induced hyperpyrexia. One systematic review of 71 cases of MDMA-induced hyperpyrexia cited a reduction in mortality from 44% to 19% when patients were given dantrolene [7]. Although this systematic review alluded to the possibility that dantrolene could also reduce the risk of severe sequelae, there have not been many other studies showing causality between the medication and reduced risks of end-organ damage in the context of MDMA overdose.

The following case series serves to further examine the role of dantrolene in the management of MDMA toxicity. Specifically, this case series focuses on the role of dantrolene in reducing the risk of end-organ damage as related to MDMA ingestion. To investigate this, we highlight the cases of 9 patients who presented to Arrowhead Regional Medical Center (ARMC) with hyperpyrexia after ingestion of MDMA at a local “rave” festival.

## 2. Methods

### 2.1. Study Population, Setting, and Data

We included patients who were admitted to Arrowhead Regional Medical Center (ARMC) between January 1, 2016, and December 31, 2018. Nine patients between the ages of 19 and 28 years of age were identified from our institution. Confirmatory testing for MDMA intoxication use was performed via urinalysis. Prisoners and individuals under the age of 18 were excluded from the study. This study was approved by the Institutional Review Board at ARMC, and researchers analyzed only deidentified data.

### 2.2. Study Definitions

Coexisting conditions were ascertained from physician documentation. Measures of end-organ damage in this study were defined by elevated creatinine (creatinine of greater than or equal to 1.5 milligrams/deciliter (mg/dL)), elevated transaminases (AST or ALT values greater than three times 40 units/liter (U/L)), elevated troponin I (troponin I > 0.3 U/L), evidence of seizures, and elevated creatine phosphokinase (CPK) levels (CPK > 1500 U/L).

## 3. Results

### 3.1. Patient Characteristics

During the period from January 1, 2016, to December 31, 2018, we identified nine patients who presented to ARMC following ingestion of MDMA in various forms including “Blue Pikachu,” “Red Winnie the Pooh,” and “Red Supreme.” The mean age was 24 (range, 19 to 28). All patients exhibited evidence of hyperpyrexia in the field following MDMA overdose. Appropriate medical evaluation and various forms of cooling measures were subsequently initiated at the event site for every patient. Seven patients received intravenous (IV) infusion of dantrolene, doses unknown, in the field in addition to external cooling measures, which included the use of ice packs and cooling blankets. All patients were orotracheally intubated due to a low Glasgow Coma Scale (GCS) score of less than 8. After initial stabilization in the field, patients were transported to ARMC's emergency department (ED). Upon arrival to the ED, patients were reevaluated and the resuscitation continued per existing protocols. Relevant labs were drawn, and patients underwent imaging studies such as chest X-ray and computerized tomography (CT) scans. All nine patients did not have any existing past medical history and were all young, healthy adults. All patients were then admitted to AMRC's medical intensive care unit for further management. Eventually, all nine patients were successfully extubated, survived, and were either discharged from the hospital or left against medical advice several days after admission. We were unable to follow these patients past their admissions in the hospital. We therefore could not monitor any trend in their lab values to determine if end-organ damage was acute or if it eventually became chronic.

### 3.2. Laboratory and Radiologic Findings

All pertinent vitals and lab values from our patients have been included in Table 1. Radiologic findings did not show evidence of acute abnormalities. All of the patients in our case series exhibited evidence of hyperpyrexia and 8 out of 9 experienced some degree of end-organ damage on arrival to the hospital. Recorded body temperatures in the field prior to cooling measures and/or dantrolene administration ranged between 102.6 F (degrees Fahrenheit) and 109.0 F. The most common forms of end-organ damage experienced by patients included acute kidney injury (66.67%), rhabdomyolysis (66.67%), and elevated troponin I (55.56%). Additionally, two patients experienced seizures, and three patients suffered from transaminitis. Six out of seven patients in the dantrolene plus cooling measure group experienced a decrease in their temperature to less than 100.0 F by the time they arrived to the hospital's ED. One of the two patients who did not receive dantrolene also had a decrease in body temperature to less than 100 F by the time they arrived to the ED. All seven of the patients who received dantrolene experienced at least one type of end-organ damage.

Furthermore, subgroup analysis (Table 2) was performed based on initial core body temperature prior to intervention. In the subgroup of five patients with an initial core body temperature of at least 108.0 F, all patients suffered from at least two forms of end-organ damage (mean number of organs damaged was 3.25). We compared this to a subgroup of patients who had initial core body temperatures of less than 108.0 F and found that only one of these four patients suffered from more than one form of end-organ damage (mean number of organs damaged was 1.5). *P* values for all data in Table 2 were >0.1. Additionally, the highest creatinine, CPK, troponin, and transaminase levels all decreased to normal limits over the hospital course in the subgroup of patients with initial core body temperatures of at least 108.0 F.

## 4. Discussion

Unlike many other toxicological emergencies, MDMA overdose lacks a concrete algorithm for management. Treatment of hyperpyrexia related to MDMA ingestion starts with supportive measures including chilled IV fluids, ice baths, and cooling blankets. Cooling measures are the gold standard for reduction of core body temperature, as prolonged hyperpyrexia is associated with end-organ damage. In the case of MDMA toxicity, standard cooling measures may not adequately reduce core body temperature at an appropriate rate, which warrants the investigation of pharmacological intervention. However, the role of pharmacologic agents in this setting has yet to be formally established. Physicians have trialed various medications in attempts to reduce morbidity and mortality as related to MDMA overdose. Case reports have documented the use of cyproheptadine [8], chlorpromazine, and more extensively, dantrolene [9–12] to help mitigate MDMA-induced hyperpyrexia. These medications have all shown varying degrees of success in reducing mortality and sequelae related to MDMA overdose. However, given the lack of clinical trials and observational studies, no single medication has been favored over the rest.

To further explore the role of pharmacological intervention in the setting of MDMA overdose, our case series focused on the use of the medication dantrolene in the prehospital resuscitation arena. Founded in the 1960s, dantrolene is a skeletal muscle relaxant which works by blocking calcium release from the sarcoplasmic reticulum of a myocyte [13]. This leads to a decrease in intracellular calcium concentrations that ultimately results in excitation-contraction decoupling and reduced muscle tone. A reduction in muscle tone translates into a lack of heat production, which can then lead to a decrease in core body temperature in a variety of clinical contexts. Common adverse effects of dantrolene include drowsiness, dizziness, weakness, fatigue, and diarrhea. While the mechanism of action makes theoretical sense, the clinical application of dantrolene has been slightly more controversial. In a review by Hadad et al., animal and human subjects with elevated body temperatures or clinically defined heat stroke were given dantrolene; the results demonstrated a mixed picture with either an increase or no change in cooling rate [14]. The largest human study from the review (*n* = 52) showed dantrolene did not increase the rate of cooling when added to evaporative cooling techniques in heat stroke patients [15]. Therefore, the role of dantrolene in clinical practice has historically been called into question.

Our investigation centered on the previous hypothesis that early administration of dantrolene would rapidly reduce core body temperature and thereby reduce the risk of severe sequelae in MDMA overdose [7]. We identified seven cases of MDMA-related hyperpyrexia that were treated with both dantrolene and cooling measures in the field and two cases that were only treated with cooling measures. Since hyperpyrexia is associated with end-organ damage, the most optimal time to administer dantrolene would be upon presentation to medical care. Our patients all received variable doses of intravenous dantrolene by emergency medical services and were immediately transferred to our hospital for higher level of care. While almost all patients in the dantrolene plus cooling measure group experienced a rapid drop in their temperatures, it is difficult to attribute this temperature correction specifically to the use of dantrolene alone due to the small sample size of patients who did not receive dantrolene and the observation that patient 6 had a reduction in body temperature to less than 100.0 F without dantrolene administration. Furthermore, despite the early administration of dantrolene in the field, all patients who received the medication suffered from some form of end-organ damage. Although these patients went on to survive, it appears that dantrolene had no overall effect on the rate of end-organ damage. Dantrolene may have contributed to the rapid correction of body temperature in patients who received the medication, but this did not appear to correlate with any reduction in the overall degree of end-organ damage.

Of the group of patients transported to ARMC, the one patient who did not experience end-organ damage (patient 6 in table 1) did not receive dantrolene in the field. This was also the patient with the lowest initial core body temperature (102.6 F). It is therefore possible that initial core body temperature is a better predictor of end-organ damage than the degree of temperature reduction precipitated by medications like dantrolene. This theory is supported by one systematic review on MDMA overdose which delineated patients into categories based on their initial core temperature [7]. In this systematic review, it was noted that patients with initial core temperatures of 40.0 to 41.9 C (104.0 F to 107.4 F) had a higher number of both severe and mild-to-moderate complications in comparison to the group of patients with initial temperatures of 38.0 to 39.9 C (100.4 F to 103.8 F), irrespective of dantrolene administration. Our subgroup analysis supplements these findings by reflecting more forms of end-organ damage in patients with core body temperatures of ≥108.0 F (mean number of organs damaged = 3.2) when compared to patients with core body temperatures of <108.0 F (mean number of organs damaged = 1.5), again, irrespective of dantrolene administration.

The highest degree of end-organ damage was observed in the group of five patients who presented with temperatures of at least 108 F, irrespective of dantrolene administration. In the subgroup of patients with body temperatures of at least 108 F, the one patient who did not receive dantrolene experienced damage to 3 organs, while the three patients who received dantrolene experienced damage to a mean of 3.25 organs. From this limited data, it appears that dantrolene did not have a significant role in the reduction of end-organ damage. Rather, our observations, although statistically insignificant, support the theory that sequelae from MDMA overdose are more likely a function of the degree of initial hyperpyrexia.

The primary limitation to our study is that our sample size was small, and this is due to the dearth of patient cases of MDMA-induced hyperpyrexia noted at our institution, as it is a relatively rare disease process. Although we cannot draw definitive conclusions based on a sample size of nine patients, our results support the findings of Grunau et al. and provide preliminary evidence that may aid in future studies that assess the efficacy of dantrolene administration on end-organ damage in the setting of MDMA overdose. In the future, a multi-institutional study may produce a larger sample size to better quantify and statistically analyze the efficacy of this pharmacologic agent. A second limitation includes a lack of data regarding the dosing of dantrolene provided to our patients in the field. Future studies may attempt to correlate morbidity and mortality with the dosing and route of dantrolene administration provided to patients suffering from MDMA overdose.

## 5. Conclusions

The purpose of this case series was to provide more preliminary data regarding the use of dantrolene in the setting of MDMA overdose and MDMA-induced hyperpyrexia. Many case reports and a number of systematic reviews have previously linked possible improved mortality with the administration of dantrolene in this setting. However, few case reports and systematic reviews have specifically touched upon the subject of morbidity. Our case series showed no significant improvement or prevention of end-organ damage when dantrolene is provided to patients with MDMA-induced hyperpyrexia. We believe that the degree of end-organ damage suffered by patients with MDMA intoxication is more likely a function of initial core body temperature, irrespective of dantrolene administration. Future studies would benefit from having a larger sample size and including a larger number of patients who did not receive dantrolene prior to admission into the hospital, as this would be ideal for comparative purposes. Additionally, further analyses of a larger sample size can be performed to determine the optimal dosing of dantrolene in the setting of MDMA toxicity.

## Figures and Tables

**Table 1:** All patient data for dantrolene administration, body temperatures, serum creatinine, serum sodium, serum AST, serum ALT, serum troponin, serum CPK, evidence of seizures, evidence of end-organ damage, number of organs affected, and mortality.

Patient	Dantrolene (yes/no)	Temp (F) in field	Temp (F) ED arrival	Cr (mg/dL)	Sodium (mEq/L)	AST (U/L)	ALT (U/L)	Troponin (U/L)	CPK (U/L)	Seizure (yes/no)	End-organ damage (yes/no)	Number of organs affected	Mortality
Patient 1	Yes	106	99.5	1.7	138	44	48	<0.3	664	No	Yes	1	None
Patient 2	No	108	102.9	2.7	144	79	19	0.63	65730	No	Yes	3	None
Patient 3	Yes	106	95.7	2.4	137	195	293	2.27	1311	Yes	Yes	4	None
Patient 4	Yes	108	104	2.2	130	413	512	0.95	41262	No	Yes	4	None
Patient 5	Yes	109	88.1	1.6	138	29	20	2.56	60669	Yes	Yes	4	None
Patient 6	No	102.6	97	1.1	138	78	52	<0.3	473	No	No	0	None
Patient 7	Yes	108.3	89.9	1.5	137	40	49	0.45	2553	No	Yes	3	None
Patient 8	Yes	107.5	93.3	1.1	137	64	40	<0.3	6408	No	Yes	1	None
Patient 9	Yes	108.3	96.1	1	133	306	296	<0.3	11332	No	Yes	2	None

Legend: F: Fahrenheit; mEq/L: milliequivalents per liter; mg/dL” milligrams per deciliter; U/L: units per liter; Cr: creatinine; AST: aspartate transaminase; ALT: alanine transaminase; CPK: creatine phosphokinase.

**Table 2:** Comparison of variables between the initial core body temperature groups (<108 F vs. ≥108 F).

	Initial core body temperatures < 108 F (*n* = 4)	Initial core body temperatures ≥ 108 F (*n* = 5)
Creatinine (mg/dL)	1.4 (1.1, 2.05)	1.6 (1.5, 2.2)
Sodium (mEq/L)	137.5 (137, 138)	137 (133, 138)
AST (U/L)	71 (54, 136.5)	79 (40, 306)
ALT (U/L)	50 (44, 172.5)	49 (20, 296)
Troponin levels (U/L)	2.27 (0, 1.135)	0.63 (0.45, 0.95)
CPK (U/L)	987.5 (568.5, 3859.5)	41262 (11332, 60669)
Mean number of organs affected	1.5 (0.5, 2.5)	3.2 (2.5, 4)
Number of patients with end-organ damage	3	5
Mortality	0	0

Legend: F: Fahrenheit; mEq/L: milliequivalents per liter; mg/dL: milligrams per deciliter; U/L: units per liter; Cr: creatinine; AST: aspartate transaminase; ALT: alanine transaminase; CPK: creatine phosphokinase.

## Data Availability

The authors confirm that the data supporting the findings of this study are available within the article. All data in our study were obtained from our hospital's electronic medical record system. Any inquiries regarding supporting data availability of this study should be directed to the corresponding author.
